# Anatomy and Biomechanical Properties of the Plantar Aponeurosis: A Cadaveric Study

**DOI:** 10.1371/journal.pone.0084347

**Published:** 2014-01-02

**Authors:** Da-wei Chen, Bing Li, Ashwin Aubeeluck, Yun-feng Yang, Yi-gang Huang, Jia-qian Zhou, Guang-rong Yu

**Affiliations:** Department of Orthopaedics, Tongji Hospital, Tongji University School of Medicine, Shanghai, China; University of South Australia, Australia

## Abstract

**Objectives:**

To explore the anatomy of the plantar aponeurosis (PA) and its biomechanical effects on the first metatarsophalangeal (MTP) joint and foot arch.

**Methods:**

Anatomic parameters (length, width and thickness of each central PA bundle and the main body of the central part) were measured in 8 cadaveric specimens. The ratios of the length and width of each bundle to the length and width of the central part were used to describe these bundles. Six cadaveric specimens were used to measure the range of motion of the first MTP joint before and after releasing the first bundle of the PA. Another 6 specimens were used to evaluate simulated static weight-bearing. Changes in foot arch height and plantar pressure were measured before and after dividing the first bundle.

**Results:**

The average width and thickness of the origin of the central part at the calcaneal tubercle were 15.45 mm and 2.79 mm respectively. The ratio of the length of each bundle to the length of the central part was (from medial to lateral) 0.29, 0.30, 0.28, 0.25, and 0.27, respectively. Similarly, the ratio of the widths was 0.26, 0.25, 0.23, 0.19 and 0.17. The thickness of each bundle at the bifurcation of the PA into bundles was (from medial to lateral) 1.26 mm, 1.04 mm, 0.91 mm, 0.84 mm and 0.72 mm. The average dorsiflexion of the first MTP joint increased 10.16° after the first bundle was divided. Marked acute changes in the foot arch height and the plantar pressure were not observed after division.

**Conclusions:**

The first PA bundle was not the longest, widest, or the thickest bundle. Releasing the first bundle increased the range of motion of the first MTP joint, but did not acutely change foot arch height or plantar pressure during static load testing.

## Introduction

The plantar aponeurosis (PA) originates from the calcaneal tubercle and extends to the forefoot. The aponeurosis consists of a medial, central and lateral part. The medial and lateral parts attach to the abductor hallucis and the musculus abductor digiti quinti pedis, respectively. These parts are usually categorized as “fascia”. The central part is thicker and is considered an “aponeurosis” [1]. As the central aponeurosis extends towards the forefoot, it divides into five separate bundles. These bundles radiate towards and attach through the plantar plates to the proximal phalanges [1]–[3]. Most anatomic studies of the PA have focused on its attachment to the calcaneus. Detailed descriptions of each central PA bundle are rare.

There is dorsiflexion of the metatarsophalangeal (MTP) joints during walking. The PA tightens via a windlass mechanism first described by Hicks [3]. All five bundles contribute to raising the foot arch. It is not known whether dysfunction of only one central bundle could affect this mechanism.

The PA and osseous longitudinal arch of the foot form a triangular truss. When the foot is weight bearing, the PA prevents the foot arch from separating and collapsing. Resection of the PA removes this supportive effect. However, proximal plantar fasciotomy is widely used to treat recalcitrant plantar fasciitis [4], [5]. The biomechanical function of the main body of the PA has been well described, but the function of the central PA bundles has not [6]–[8]. The biomechanical effect of releasing one central PA bundle is not clear.

We performed anatomic and biomechanical measurements to determine if releasing the first central PA bundle would increase dorsiflexion of the first MTP joint, lower the longitudinal arch of the foot, and change forefoot pressures.

## Materials and Methods

### Ethics Statement

This work complied with the Helsinki Declaration related to research carried out on human subjects. Ethical approval was obtained from the Human Research Ethics Committee, Tongji University School of Medicine, Shanghai, China. Written informed consent from the donor or the next of kin was obtained for research use of the patient limbs.

### Anatomic Measurement

**Figure 1:**
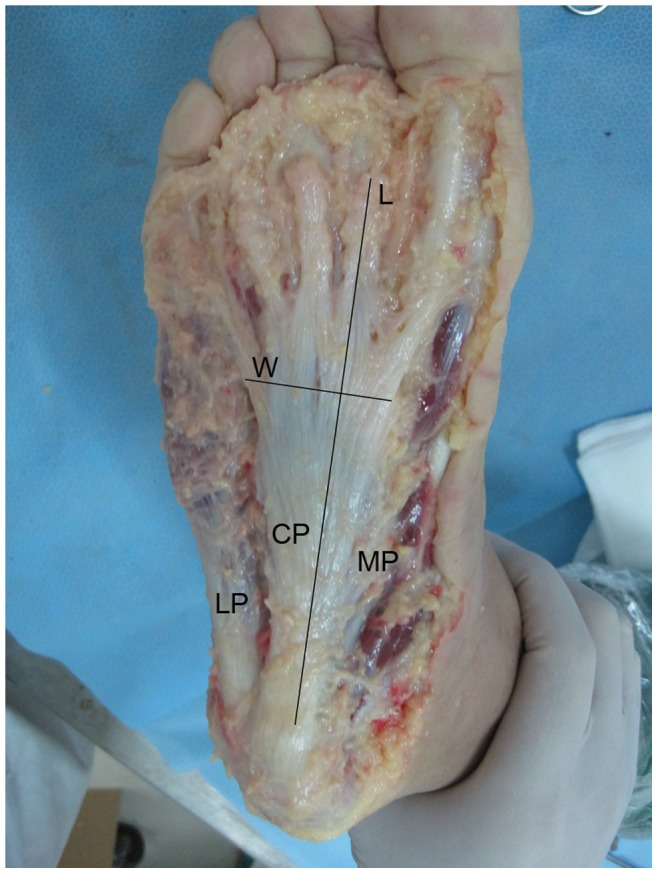
Axial view of the plantar aponeurosis. LP, lateral part; CP, central part; MP, medial part; L, length; W, width.

**Figure 2:**
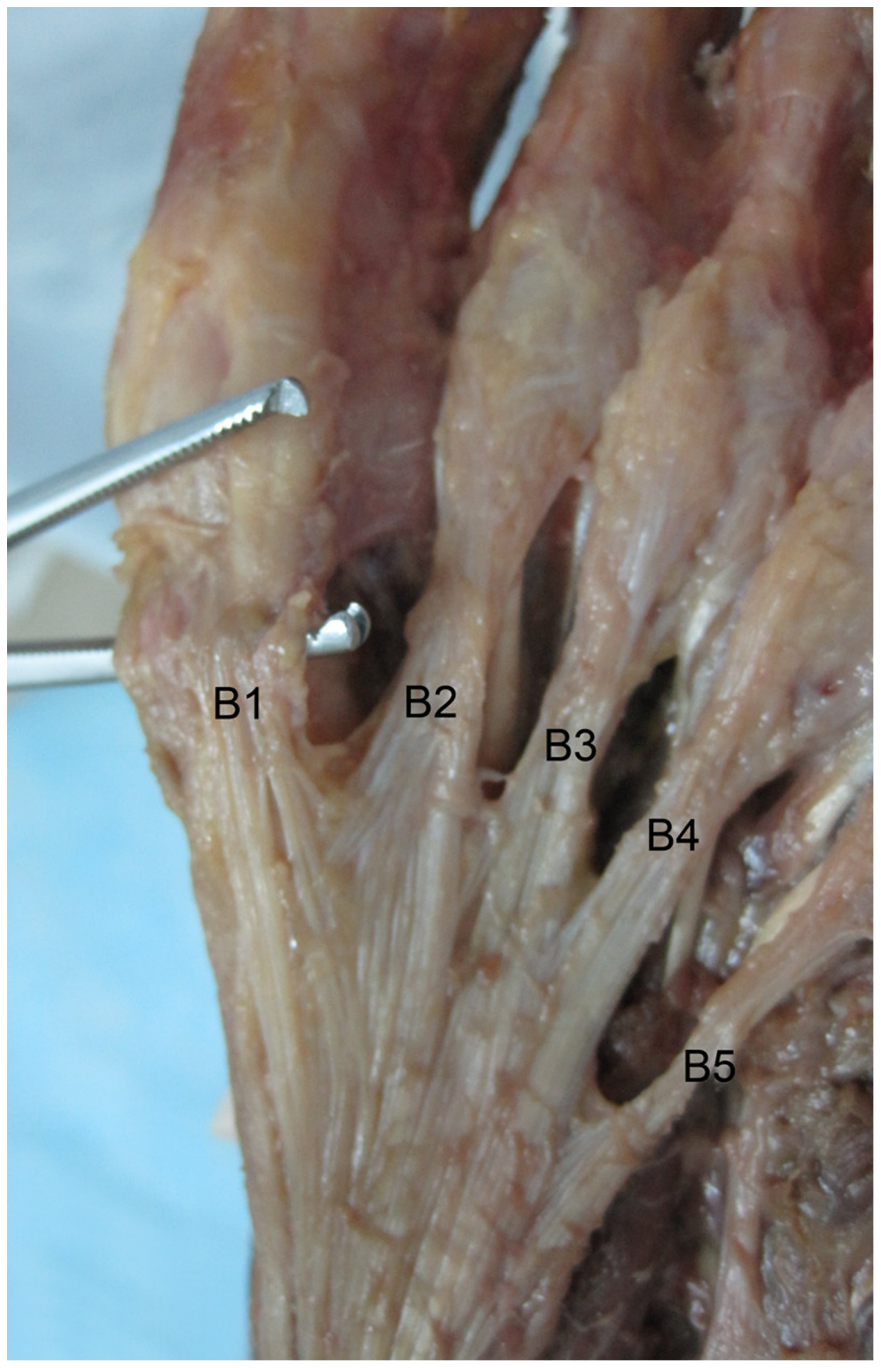
Five central part plantar aponeurosis bundles. B, bundle.

Eight fresh-frozen cadaveric foot specimens were examined. Obvious preexisting foot abnormalities were excluded by visual inspection and review of the medical history. Radiographs were performed to exclude osteoarthritis, previous fractures, tumors, osteonecrosis, and foot deformities. The mean age of the donors at death was 43.6 years (range: 32–65). The specimens were dissected through a longitudinal incision from the heel to the forefoot. The insertion of the PA to the calcaneal tubercle was exposed. The skin and soft-tissue were removed along the PA. The PA formed a complex network just distal to the metatarsal heads with superficial fibers inserting into the dermis. Only the width and thickness of the origin of each central bundle, which is at the bifurcation of the PA into bundles, were measured. The plantar plate is a fibrocartilaginous structure at the plantar aspect of the MTP joint. It is regarded as the most distal insertion of the plantar aponeurosis. The length of each central bundle was defined as the distance between the plantar plate and the origin of the bundle. Because of the different sizes of the specimens, the ratios of the length and width of each central bundle to the length and width of the central part of the PA were used to describe these bundles (Figure 1, Figure 2). The distance between the plantar plate of the second MTP joint and the origin of the central part attaching to the calcaneal tubercle was defined as the length of the central part of the PA. The maximum width of the main body of the central part, proximal to the origin of the bundles, was taken as the width of the central part. The width and thickness of the central part at its origin on the calcaneal tubercle was also measured. Each measurement was performed by three different persons using the same vernier caliper. A one-way analysis of variance (ANOVA) was used to test for differences of length of the five bundles. A Kruskal-Wallis test was used to test for differences of width and thickness of the five bundles because of inequality of variances. Post-hoc multiple comparisons were done with a non-parametric Mann-Whitney-U rank sum test when a Kruskal-Wallis test was significant. A Bonferroni-corrected value of P<0.005 was considered statistically significant. Ten comparisons were made for each anatomic parameter. Data was analyzed using SPSS software (version 14.0, Chicago, IL, USA).

### Biomechanical Range-of-motion Testing

Six specimens were collected for measuring the range of motion of the first MTP joint. The mean donor age was 38.7 years (range: 24–50). The rigid-body kinematics theorem was used in the measurement [9]–[10]. The first metatarsal and the proximal hallux were marked with three pins, as reported by Panchbhavi *et al.* [11]. The coordinate system was constructed after the specimen was fixed to the homemade braces. The y axis was the vertical projection of the long axis of the first metatarsal in the horizontal plane with the direction from proximal to distal. The x axis was perpendicular to the y axis in the coronal plane, with the direction from medial to lateral. The z axis was perpendicular to the horizontal plane with the direction determined by the right (upward) or left (downward) foot. The initial position for the measurement was the neutral position. The first MTP joint was dorsiflexed with a 10 N pull force on the distal hallux, before and after releasing the first central PA bundle. The coordinate figures of the mark points were measured using a Microscribe 3D digitizer (G2X, Immersion Corp., San Jose, CA, USA). All specimens were measured three times by the same person using the same instrument. Motion was analyzed using the Euler-Cardan approach programmed using MATLAB software (version 7.11, MathWorks, Natick, MA, USA) [12]. A paired t-test was used to compare dorsiflexion before and after the first central PA bundle was divided. A value of P<0.05 was considered statistically significant.

### Biomechanical Foot Arch and Plantar Pressure Testing

**Figure 3:**
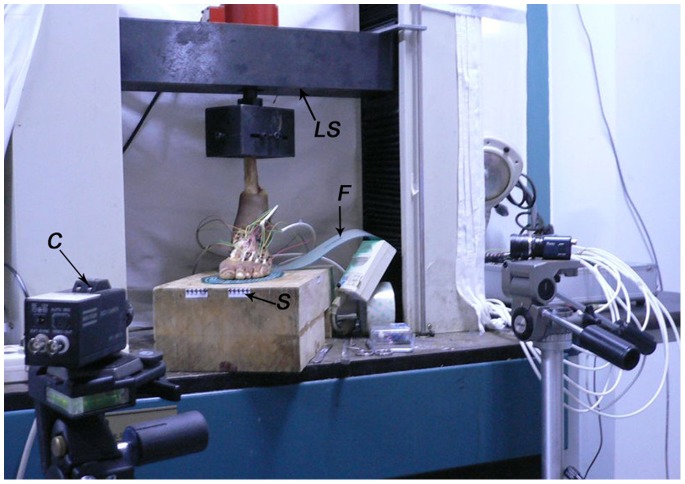
Experiment setup. LS, loading system; F, F-scan film; C, camera; S, scale.

**Figure 4:**
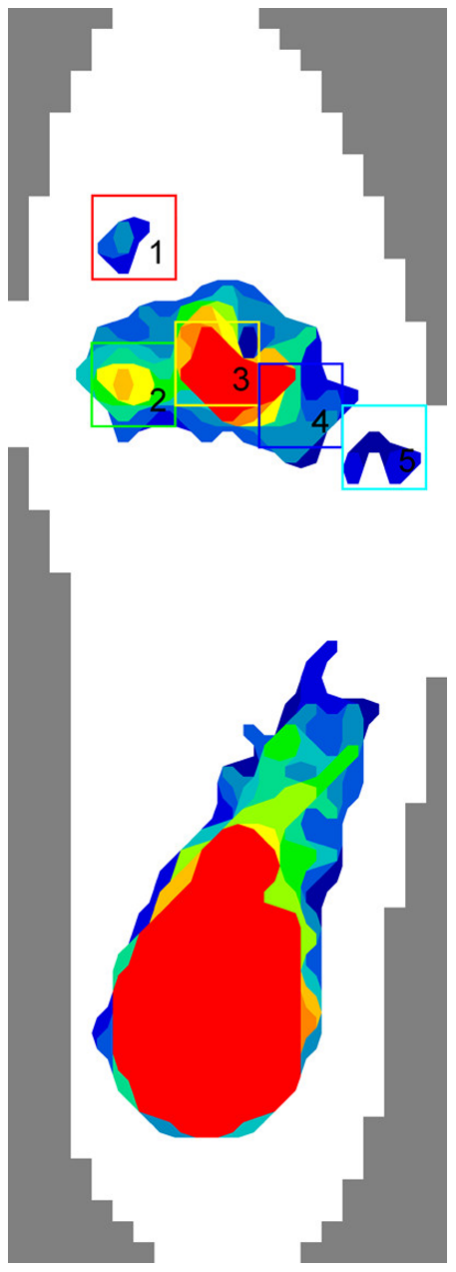
Plantar pressure distribution areas. Zone 1, hallux; Zone 2, first metatarsal head; Zone 3, second metatarsal head; Zone 4, third to fourth metatarsal head; Zone 5, fifth metatarsal head.

Another six specimens were used for the next biomechanical testing. The mean donor age was 40.2 years (range: 28–63). Each specimen was dissected to remove the dorsal skin and muscles while the ligaments were kept intact. Custom-made black-beaded pins were inserted into the navicular, cuboid, three cuneiforms and five metatarsals as identification points. Two digital cameras positioned laterally and in front of the specimen, 90° to each other, were used to capture the motion of the specimen (Figure 3). The specimen was subjected to an axial load of 600 N, equivalent to the body weight of a 60-kg subject. The axial load was applied by a universal testing machine (CSS-44010; CRIMS Co., Ltd., Changchun, China). Displacement of the identification points on the photographs was measured using an image analyzing software (Image J, version 1.46, NIH, USA) [13]. A scale on the loading platform was used to calculate actual displacement. Displacements of the navicular, medial cuneiform and first metatarsal in the inferior-superior direction, and displacements of the heads of the five metatarsals in the medial-lateral direction were measured. An F-scan plantar pressure analysis system (Tekscan Inc., Boston, MA, USA) was used to measure plantar pressure during loading. An insole sensor film containing 960 independent sensing regions was placed between the plantar foot and the loading platform. The data collection period was scheduled for 8 s, with a collection frequency of 50 frames/s [14]. Five boxes added to the plantar pressure distribution areas were used to collect the peak plantar pressure of the hallux, first metatarsal head, second metatarsal head, third to fourth metatarsal heads, and fifth metatarsal head (Figure 4). Testing consisted of four sequential procedures: (1) Without load. The arch height of the foot was measured with the MTP joints passively dorsiflexed by 45° using a wedge shaped block with an angle of 45° placed under the toes. (2) Axial load of 600 N. The displacement and plantar pressure were measured. (3) The first procedure was repeated with the first central bundle divided. (4) The second procedure was repeated with the first central bundle divided. A paired t-test was used to compare the results before and after releasing the first bundle. Statistical significance was defined as P<0.05.

## Results

Anatomic measurements were performed in eight specimens. The width and thickness of the origin of the central part of the PA at the calcaneal tubercle were 15.45±0.72 mm and 2.79±0.14 mm, respectively. The length of the central part was 146.45±9.54 mm and the width was 26.85±3.05 mm. The first bundle was not the longest, widest, or thickest of the five central bundles (Table 1). The widths (H = 22.341, P = 1.71E-4) and thicknesses (H = 26.377, P = 2.66E-5) of the five central bundles were significantly different. The lengths (F = 1.870, P = 0.138) were not significantly different. The first central bundle was wider than the fourth (Mann-Whitney U = 4, P = 0.003) and fifth (Mann-Whitney U = 2, P = 0.002) bundles, but not the second (Mann-Whitney U = 31, P = 0.916) or third bundle (Mann-Whitney U = 24, P = 0.401). The first central bundle was thicker than the third (Mann-Whitney U = 5, P = 4.57E-3), fourth (Mann-Whitney U = 0, P = 0.001), and fifth (Mann-Whitney U = 0, P = 0.001) bundles, but not the second bundle (Mann-Whitney U = 14, P = 0.059).

**Table 1:** Central aponeurosis bundle measurements (Mean ± SD, n = 8).

Bundle	L (mm)	W (mm)	T (mm)	LB/LC	WB/WC
1	42.12±5.99	7.12±2.14	1.26±0.24	0.29±0.04	0.26±0.05
2	44.43±8.41	6.68±1.39	1.04±0.22	0.30±0.05	0.25±0.03
3	41.43±6.95	6.17±0.77	0.91±0.10	0.28±0.04	0.23±0.03
4	36.48±4.42	4.93±0.21	0.84±0.07	0.25±0.02	0.19±0.02
5	40.09±3.10	4.55±0.47	0.72±0.07	0.27±0.03	0.17±0.02

NOTE: SD = standard deviation, L = length, W = width, T = thickness, LB/LC = ratio of the length of the bundle to the length of the central part, WB/WC = ratio of the width of the bundle to the width of the central part.

Dorsiflexion of the first MTP joint increased by 10.16±2.10° after releasing the first PA bundle (Table 2). The increase was statistically significant (paired t-test, t = 11.83, P = 7.59E-5).

**Table 2:** Dorsiflexion of the first MTP joint.

Cadaver	Intact(degrees)	Release FB(degrees)	Increase(degrees)
1	70.57	77.85	7.28
2	78.31	87.74	9.43
3	75.52	86.11	10.59
4	69.59	81.10	11.51
5	77.45	90.72	13.27
6	82.60	91.49	8.89
Mean	75.67	85.84	10.16
SD	4.92	5.40	2.10

NOTE: MTP = metatarsophalangeal, FB = first bundle, SD = standard deviation.

The vertical displacement of the medial cuneiform decreased significantly after the first PA bundle was released with the MTP joints dorsiflexed by 45°. The navicular and first metatarsal base had no change in vertical displacement (Table 3). No change in arch height (Table 4) or width of the forefoot (Table 5) was seen when an axial load of 600 N was placed on specimens that had the first bundle divided. The peak plantar pressure of each metatarsal heads also did not change significantly after the first bundle was divided (Table 6).

**Table 3:** Vertical displacement with the MTP joints dorsiflexed by 45° (n = 6).

Bone	Intact (mm)	Release (mm)	t value	P value
Na	−3.29±0.53	−3.21±0.57	1.52	0.19
Cu	−2.49±0.48	−2.24±0.37	2.96	0.03
MT	−1.62±0.22	−1.57±0.16	1.44	0.21

NOTE: MTP = metatarsophalangeal, Na = navicular, Cu = medial cuneiform, MT = metatarsal, “−” means upward.

**Table 4:** Vertical displacement with an axial load of 600(n = 6).

Bone	Intact (mm)	Release (mm)	t value	P value
Na	4.10±0.27	4.14±0.25	0.86	0.43
Cu	3.46±0.24	3.49±0.17	0.73	0.50
MT	2.48±0.20	2.53±0.21	1.37	0.23

NOTE: Na = navicular, Cu = medial cuneiform, MT = metatarsal.

**Table 5:** Transverse displacement of the metatarsal head with an axial load of 600(n = 6).

Bone	Intact (mm)	Release (mm)	t value	P value
MT 1	−1.48±0.11	−1.50±0.13	1.17	0.30
MT 2	1.24±0.10	1.25±0.10	0.67	0.53
MT 3	1.54±0.12	1.55±0.17	0.36	0.74
MT 4	1.87±0.18	1.91±0.20	1.03	0.35
MT 5	2.04±0.16	2.03±0.14	0.25	0.81

NOTE: MT = metatarsal head, “−” means medial.

**Table 6:** Peak forefoot plantar pressure with a 600-N load (n = 6).

Bone	Intact (KPa)	Release (KPa)	t value	P value
P1	13.83±3.76	12.50±3.08	1.06	0.34
MT1	33.17±6.97	32.33±6.95	0.47	0.66
MT2	67.17±6.43	69.50±5.09	0.94	0.39
MT3–4	58.17±6.31	59.50±5.54	0.69	0.52
MT5	21.67±5.13	20.50±3.94	0.67	0.53

NOTE: P = phalange, MT = metatarsal head.

## Discussion

The anatomy of the PA has been well described in the literature, but most descriptions focused on the attachment of the PA to the calcaneus. This is the first study to give a detailed measurement of all five central PA bundles. Since the number of specimens was limited, it cannot be proved that the first bundle was the strongest bundle. The first bundle was not the longest, widest, or thickest of the five central bundles. Further biomechanical testing of the failure load of each bundle is needed [15]. The thickness and width of the origin of the central part of the PA we measured were similar to previous reports [16], [17]. The PA is often thickened greater than 4 mm in plantar fasciitis [18]. Our measurements reflected the normal anatomy of the PA. The lateral part of the PA we evaluated was much thicker than the medial part. The medial part was very thin and could not be easily measured. The color of the lateral part was white, similar to the central part. But the medial part was almost transparent. Some avulsion fractures occurring at the proximal end of the fifth metatarsal are caused by forces directed through the lateral part, which indicates that the lateral part is very strong [19]. Based on these findings, we think it is more appropriate to categorize the lateral part as an “aponeurosis” and the medial part as a “fascia”, an opinion which differs from that of Aquino *et al.* [1].

One function of the PA is to support the foot arch. The effect of dividing the PA on the foot arch has been extensively investigated. Murphy *et al.* [7] used cadaveric specimens to evaluate changes in the medial and lateral longitudinal arches after sequential division of the PA. Complete release of the PA resulted in greater loss of arch height than release of the medial third part. There was 62% less medial arch and 100% less lateral arch. A biomechanical model constructed by Arangio *et al.* [6],using a load of 683 N applied to a foot with the PA divided, demonstrated a 17% increase in vertical displacement and 15% increase in horizontal elongation. Thordarson *et al.* [8] also found a consistent decrease in arch support with sequential division of the PA in eight cadaveric specimens. Almost all related studies used a technique where the main body of the central part of the PA was released. The biomechanical effect of releasing only one PA bundle has not been reported. We found that releasing the first bundle did not acutely lower the foot arch when an axial load of 600 N was applied. This indicates that the arch support was not weakened by resection of the first bundle.

The windlass mechanism of the PA was examined. The MTP joints were dorsiflexed 45° to test the elevating effect on the foot arch. Releasing the first bundle did not acutely weaken the elevating effect. The upward displacement of the navicular and first metatarsal base did not decrease significantly after dividing the first bundle. This indicates that the arch-raising effect was preserved by the remaining four bundles. During dissection, we noticed that some plantar muscles (flexor digitorum brevis) were firmly attached to the PA. We suspect these muscles might also contribute to elevating the foot arch when the PA is tightened by extending the toes. As a result, the arch-raising effect was maintained after dividing the first bundle.

The transverse displacements of the metatarsal heads were measured to identify the effect of releasing the PA on the forefoot. With the axial load applied, the width of the forefoot increased, allowing medial movement of the first metatarsal head and lateral movement of other four metatarsal heads. Releasing the first PA bundle did not cause the width of the forefoot to change significantly, similar to the findings of Waldecker *et al.* [20]. With the specimens axially loaded to 900 N, they found that the overall effect of PA release on the structure of the forefoot was not significant. However, they performed a proximal plantar fasciotomy, while we only divided the first bundle. Dividing the PA may not affect forefoot width if the integrity of the intermetatarsal ligaments is maintained.

The effect of releasing the first central bundle on forefoot plantar pressure was also evaluated. The plantar pressure of the forefoot did not change significantly after the first bundle was divided when an axial load of 600 N was applied to the foot. These results were different from other studies where the PA was released next to the calcaneus. Sharkey *et al.* [21] found an increase of plantar pressure under the metatarsal heads after releasing the medial half of the central part. Complete release resulted in a pressure shift from the toes to the metatarsal heads. Another study by Erdemir and coworkers [22] reported similar results. The site at which the release was performed might play a role in these different findings. Near the calcaneus, the central part bears relatively higher loads than in any one central bundle. Releasing the whole central part can also lead to a deformation of the foot arch. Consequently, dividing the PA near the calcaneus would change the load distribution of the forefoot. Differences in loading environments could also have contributed to the difference in findings. The test would be more meaningful if the late stance phase (when the heel is off the ground) was simulated. Unfortunately, during our preliminary tests, the foot position could not be consistently maintained with the heel off the loading platform. As a result, foot loading in the neutral position was chosen as our definitive test. A review of the literature found no study reporting how force was transmitted through each bundle or the whole central PA during gait. Although the ideal testing method has not been identified, we speculate that testing the tension forces in each bundle and the whole central part might reveal the true function of the PA and help define the role of the first bundle in foot function.

Six specimens were used to measure the change in dorsiflexion of the first MTP joint after releasing the first central bundle. Dorsiflexion increased by 10.16°, comparable to the results reported by Harton *et al.* [23]. They performed a proximal plantar fasciotomy in eighteen patients with recalcitrant plantar fasciitis in the absence of first MTP joint pathology. This resulted in an average increase of dorsiflexion in the first MTP joint of 9.8°. A load of 10 N was applied to the distal hallux to produce dorsiflexion of the first MTP joint in our testing. Although the load chosen was different from that found during walking, we think it had two advantages. First, it could be easily reproduced when different specimens were measured. Second, this load was relative small, avoiding injury to the joint. These factors served to increase the reproducibility of the testing.

Hallux rigidus is the second most common disorder of the first MTP joint next to hallux valgus [24]. Some studies suggest that a tight or short first central PA bundle may contribute to hallux rigidus [25], [26]. Davies-Colley [27] treated patients with hallux rigidus by dividing the first PA bundle and part short muscles in the sole of the foot. At present, release of the distal insertion of the first central bundle is still, but not universally, used to treat early-stage hallux rigidus [28]. The first central bundle was divided in our testing, causing increased dorsiflexion of the first MTP joint. However, the specimens had no hallux rigidus. Further studies are needed to determine whether releasing the first central bundle would benefit hallux rigidus.

There were some limitations to our study. Loading the foot in the neutral position was not consistent with normal walking. Unfortunately, simulating the late stance phase with the heel off the ground could not be reproduced by our equipment. Also, dynamic loading to simulate pressures obtained during walking could not be performed. A load of 600 N was chosen for the biomechanical testing. A much heavier load or repeated loading might produce different results. Normal specimens were used to measure the dorsiflexion of the first MTP joint. The results of our testing may not be applicable for those with hallux rigidus.

In conclusion, the first PA bundle was not the longest, widest or the thickest of the five central bundles. Releasing the first bundle increased the range of motion of the first MTP joint, but did not acutely change the arch height or plantar pressure in static tests. Further studies are needed to determine whether releasing the first central bundle would benefit hallux rigidus.
